# Leeches

**Published:** 1883-06

**Authors:** 


### Leeches.

The *Chemist and Druggist*, March 15, 1883, says the plan that seems most uniformly successful in keeping leeches is to place them in a vessel of water about half full, with about half a dozen rough stones the size of oranges, changing the water about once in six months. If the sides of the vessel get covered with vegetation, clean them down with the water, but do not change it. If a leech dies and taints the water, it must be changed. As a rule, it has merely a fishy odor. Some prefer to grow water-plants, such as *Anacharis* or *Vallisneria*, in the vessel.

A WRITER in the Medical Times declares that alcoholism is unknown in Brazil, and the cause is coffee. Cafes in which the most delicious infusions of the bean are dispensed, abound there, where saloons for malt and spirituous beverages abound here. His Excellency, the Baron of Theresopolis, Vice-Director of the faculty of medicine of Rio de Janeiro, proclaims that the number of drunkards in a country is in inverse ratio to the amount of coffee consumed.—*Medical Age.*

"FORT MIT DEM SPRAY."—Time does not in any way tend to reduce the feeling of disfavor with which employment of the spray is regarded on many hands, and lately Mr. Christopher Heath dealt another and by no means insignificant blow at the same adjunct to antiseptic surgery. To the cooling effects produced by the spray during performance of an ordinary operation, Mr. Heath unhesitatingly ascribed a very considerable part of the fatal consequences which frequently follow in such cases; nor can we feel any great surprise that such a conclusion should be entertained. It is reasonable to assume that exposure of a wounded surface for the space of half-an-hour or more to conditions such as are set us by a vaporous surrounding of the part, and constant liquefaction of steam, must be provocative of most injurious consequences by reducing local temperature. But there is a still graver charge preferable in the same connection against the spray—that, namely, it leads to absorption of carbolic acid in poisonous quantities, and so brings about one of the principal evils associated with antiseptic surgery, according to strict Listerism. The failure to detect carboluria after such operations may, as Mr. Heath pointed out, possess the utmost significance; for when the kidney does not excrete the poison, it remains to do its deadly work in the system. It may be pointed out here that it is taken for granted that carbolic poisoning ensues as a necessary consequence of using the spray—a conclusion that would seem to receive very general adoption, since quite a large proportion of strictly antiseptic operators are avoiding the dangers incurred by its employment. Dr. Andrew Clark, President of the Clinical Society, probably expressed the opinion of the profession when, after Mr. Heath's remarks, he uttered the hope that the speaker would take an early opportunity of expounding the views he had briefly drawn attention to. The subject is of such importance that we hope Mr. Heath may speedily accept the friendly challenge thrown down to him.—*The Medical Press.*

THE Medical Department of the State University of Iowa will insist in future that all applicants for admission must pass a preliminary examination in the English branches.

